# The Role of Astrocytes in the Regulation of Synaptic Plasticity and Memory Formation

**DOI:** 10.1155/2013/185463

**Published:** 2013-12-04

**Authors:** Yusuke Ota, Alexander T. Zanetti, Robert M. Hallock

**Affiliations:** Neuroscience Program, Skidmore College, 815 N Broadway, Saratoga Springs, NY 12866, USA

## Abstract

Astrocytes regulate synaptic transmission and play a role in the formation of new memories, long-term potentiation (LTP), and functional synaptic plasticity. Specifically, astroglial release of glutamate, ATP, and cytokines likely alters the survivability and functioning of newly formed connections. Among these pathways, regulation of glutamate appears to be most directly related to the promotion of LTP, which is highly dependent on the synchronization of synaptic receptors through the regulation of excitatory postsynaptic potentials. Moreover, regulation of postsynaptic glutamate receptors, particularly AMPA receptors, is dependent on signaling by ATP synthesized in astrocytes. Finally, cytokine signaling is also implicated in regulating LTP, but is likely most important in plasticity following tissue damage. Despite the role of these signaling factors in regulating LTP and functional plasticity, an integrative model of these factors has not yet been elucidated. In this review, we seek to summarize the current body of evidence on astrocytic mechanisms for regulation of LTP and functional plasticity, and provide an integrative model of the processes.

## 1. Introduction

The long-term storage of information in the form of memory is one of the principal functions of the developed nervous system. The ability to utilize this information provides evolutionary advantages in adapting and responding to situations in a given environment. The method for the formation of memories and the process of functional specialization in the brain during development has been found to be mediated by both structural and functional plasticity, including long-term potentiation between neurons [1]. While much attention has been given to these processes on a neuronal level, less attention has been given to what role glial cells, particularly astrocytes, may have in the underlying mechanisms.

While astrocytes were formerly thought to serve mostly as housekeeping cells, they have recently gained attention as an integral part of the chemical synapse. In addition to their structural and metabolic roles, astrocytes are now thought to be heavily involved in synaptogenesis and in regulating the communication between already formed connections [2]. Several studies have demonstrated that astrocytes utilize both ionotropic and metabotropic systems in order to regulate neuron to neuron communication [3–5], and that they may have specific mechanisms for regulating the formation of memories. Here, we review recent evidence for the importance of astrocytes in both structural and functional synaptic plasticity, specifically long-term potentiation, the key chemical transmitters that are involved (Table 1), as well as the underlying mechanisms by which astrocytes may regulate these processes.

## 2. Glial Cells: Astrocytes

Glial cells are nonneuronal cells that are now believed to constitute 50% of the cells in the whole brain in humans and other primates [6–9], although other reports have suggested that glia may outnumber neurons 10 : 1 [10–12]. Astrocytes, as their name suggests, appear to be star-shaped when Golgi stained or immunostained for glial fibrillary acidic protein [5]. However, the morphology and physiology of astrocytes differ depending on the type [13]. Typically, astrocytes have a complex structure that is highly branched with many small protrusions that contact the synaptic cleft [14, 15]. With their unique morphology, astrocytes form the blood brain barrier, have a role in ion homeostasis, and form the tripartite synapse [16]. The blood brain barrier is made up of capillary endothelial cells, vascular pericytes, and the perivascular endfeet of astrocytes. Together, they create a highly selective barrier that allows oxygen and hormones to permeate into the brain while preventing the passage of other molecules due to possible harmful effects.

Astrocytes also maintain homeostasis of various ions such as sodium, potassium, chloride, and hydrogen [17]. For instance, astrocytes play a critical role in regulating extracellular K^+^ levels. When the extracellular concentration of K^+^ is high, astrocytes uptake the ion using transporters or channels and transfer it to adjacent astrocytes via gap junctions by a process called spatial buffering [18–21]. Due to this process, astrocytes prevent extracellular concentrations of K^+^ from reaching toxic levels.

In the tripartite conceptualization of the synapse, perisynaptic astrocytes are present along with the standard presynaptic and postsynaptic neurons [15, 22–24]. Contact made by perisynaptic astrocytes with the synaptic cleft depends on the type and location of synapses [13, 15, 25]. In the hippocampus for instance, 64% of synapses are contacted by perisynaptic astrocytes at the synaptic cleft [26]. The intricate arborization and ramifications of astrocytes allow them to tightly enwrap the synaptic terminal in order to modulate synaptic processes [14, 15, 25]. Previous studies suggest that astrocytes respond to neurotransmitter release by increasing their intracellular calcium levels and controlling neuronal excitability through the release of gliotransmitters [2]. Based on findings that explain the functioning of the tripartite synapse, more attention has been given to the potential role of how astrocytes aid memory. In areas known for synaptic plasticity, such as the hippocampus, astroglial membranes appear to surround the majority of larger axo-dendritic synapses, and around 60% of all synapses in the hippocampus [27, 28]. In astrocytes that are part of a tripartite synapse, calcium peaks, which correspond to calcium oscillations tuned to neuronal activity, cause the proximal and distal release of glutamate from the astrocyte to neighboring neurons [5].

Excess glutamate is taken up by astrocytes and further regulated through a shunting cycle by which it is broken down into glutamine, repackaged, sent to the presynaptic-neuron, and finally converted back into glutamate [16]. Astroglial glutamatergic regulation is so widespread that it is estimated that only 20% of synaptic glutamate is taken up by transporters on the postsynaptic neuron, while the other 80% is processed by transporters such as the glutamate aspartate transporter (GLAST) on the membrane of the associated astrocyte [29]. Additionally, astrocytes have the ability to swell and shrink in size through the use of aquaporin channels, and this may allow them to reduce the leakage of neurotransmitters, increasing the active concentration in the synapse, and preventing spillover in the case of damage [30, 31]. However, transmitters can also be released through these channels when exposed to a hypotonic bath solution, ischemia, or a traumatic brain injury [32–34]. Besides their role in signaling, astrocytes have also been implicated in controlling the development of the nervous system through factors such as axon guidance and synaptogenesis, as discussed below.

## 3. Plasticity in the Hippocampus

It is now well known that the hippocampus, located in the inferior temporal lobe, is responsible for the formation and storage of memory [35, 36]. The hippocampal structure has distinct functional areas implicated in memory formation, that is, the CA1, CA3, and the dentate gyrus. Various parts of the brain display some form of synaptic plasticity, but the hippocampus is one of the structures that has received much attention due to its overall functional importance.

Synaptic plasticity refers to experience mediated structural and functional changes to the connections between neurons that results in changes to neural circuits [37–39]. These neural circuits are often developed (synaptogenesis) and strengthened through the reinforcement of some connections and the removal of others (synaptic stripping), which can occur in response to environmental experience. During early development, plasticity can occur through large scale dendritic and axonal conformational changes, and while these processes are observed in the adult mammalian brain, the scale on which they take place and the efficacy of regulatory processes involved are inhibited [40]. While less overall change is observed in the adult brain, early developmental plasticity in children, as well as memory formation and learning in adults, are both likely dependent on structural changes in the functioning of the synapse itself [41–43]. Recent evidence shows that even small structural changes to the dendritic spines can drastically alter the overall output/input of synaptic protein receptors, which in adults is likely more important in determining neuronal activity than dendritic spine density [41–43]. Due to a high concentration of synapses in the brain, tuning of activity could be accomplished through regulating synaptic function with relatively little conformational change, which is important in being able to learn and store large quantities of information without negatively impacting other signaling pathways.

The tuning of synaptic activity associated with functional plasticity, or changes in synaptic strength [44], has been demonstrated through the modulation of membrane receptors by enzymatic activity such as phosphorylation [39, 45–47]. However, changes to the chemical environment within the synapse are likely more influential in the associated changes to neuronal firing and receptor concentrations. The changes in dendritic spines and synaptic activity in relation to plasticity have been found to be most closely linked to synaptic glutamate receptors and changes in both internal and external calcium in neurons [48]. It is consistently demonstrated that neuron-glia interactions are essential to this type of environmental regulation, with astrocytes being paramount in regulating signaling molecules such as glutamate which are particularly important in the plasticity and learning processes [49, 50]. As astrocytes are in part often responsible for regulating synapse formation and synaptic activity, there is a strong possibility that their activity plays an integral role in plasticity and learning.

Among the various forms of functional synaptic plasticity, long-term potentiation (LTP) has received much attention in the hippocampus due to its role in memory [51]. LTP is the process of a long-lasting enhancement in synaptic strength. This was first observed in electrophysiological studies, using high-frequency stimulation (100 Hz) of neurons in the perforant pathway and recording the activity at the dentate gyrus [52]. Electrode recordings followed by tetanic stimulation exhibited a longer lasting excitatory postsynaptic potential (EPSP) of the postsynaptic neuron in the dentate gyrus. The mechanism of LTP differs depending on the location of the hippocampus. For example, N-methyl D-aspartate (NMDA) receptor dependent LTP occurs at the Schaffer collateral region while NMDA receptor independent LTP is observed at the mossy fibers of the CA3 region [53–57]. Despite various forms of LTP that occur in the hippocampus, NMDA receptor dependent LTP is heavily studied. Several studies blocking NMDA receptor activity showed impairment in different types of memory in mice, implicating NMDA receptors in memory formation [58–61]. However, these studies do not indicate that LTP causes memory, as LTP may be an underlying process that helps form memory but does not directly cause it.

For NMDA receptor dependent LTP to occur, glutamate binding to NMDA receptors and depolarization of the neuron is required. Activation of NMDA receptors allows calcium to stimulate cyclic adenosine monophosphate (cAMP) release, causing a cascade of signaling mechanisms involving protein kinase A, cAMP response element binding protein (CREB), cAMP response element (CRE), mitogen activated protein kinase, and calcium calmodulin dependent protein kinase II [62–64]. These factors lead to the upregulation of transcription. Therefore, synthesis of new proteins underlies the mechanism for long-term memory. While much attention has been given to the regulation of hippocampal neurons by these factors, there is a growing body of evidence that astrocytic support is more critical in the regulation and function of many LTP related compounds and mechanisms than previously thought.

## 4. LTP Associated Gliotransmitters

Astrocytes release and regulate several neuroactive molecules that can affect neuronal activity and modulate plasticity and LTP. These compounds (summarized in Table 1) include glutamate, ATP, cytokines, and several other key signaling molecules like D-serine, adenosine, and lactate [65]. Glutamate plays a key role in the regulation of synaptic activity and causes a response in astrocytes [66, 67]. Importantly, astrocytes actively sequester up to 90% of glutamate that is released into the extracellular space between neurons [68, 69]. Glutamate causes a wide range of effects in astrocytes via metabotropic glutamate receptors (mGluR), NMDA receptors, and *α*-Amino-3-hydroxy-5-methyl-4-isoxazolepropionic acid (AMPA) receptors.

Although cortical astrocytes generally express functional NMDA receptors, this does not appear to be the case for hippocampal astrocytes [70–72]. Hippocampal astrocytes do not exhibit activation upon standard NMDA receptor agonists [70, 72]. Functional AMPA receptors, on the other hand, are expressed by hippocampal astrocytes [73, 74]. Additionally, hippocampal astrocytes change the properties of their AMPA receptors during postnatal development. At the beginning stages of postnatal development, low levels of AMPA receptor currents were observed and significantly increased around P12 [75]. Immature astrocytes also had a prolonged activation of the AMPA receptor, which induced an influx of Na^+^ and Ca^2+^. As astrocytes matured, glutamate responses greatly increased as well.

Astrocytes are likely able to synchronize with neuronal activity and subsequently regulate glutamate transmission between neurons [76–79]. For example, astrocytic glutamate release activates presynaptic NMDA receptors and promotes increased excitatory communication between neurons [80]. These NMDA receptors are also subjected to further regulation by endogenous concentrations of D-serine, which serves as a coagonist, specifically in the hippocampal region, suggesting its potential importance in new memory formation [81–84].

In addition to the ionotropic glutamate receptors, astrocytes also use mGluRs. In the hippocampus, mGluR1 [85, 86], mGluR5 [87, 88], and mGluR3 [89, 90] are expressed and functionally important in astrocytes and the modulation of neuronal activity. However, astrocytes of 1-week-old mice, but not older mice, express high levels of mGluR5 [90]. Furthermore, astrocytes of adult mice did not exhibit an increase in Ca^2+^ when stimulated with an mGluR5 agonist. These results suggest developmental changes in the expression of mGluRs in astrocytes. Contradictory to the observations by [90], there has been research demonstrating mGluR5's role in Ca^2+^ elevation in adults. Further research must be done to clarify these opposing findings.

Astrocytic activation can result in the release of gliotransmitters that can affect neuronal activity. Gliotransmitter release is Ca^2+^ dependent and involves the following mechanism. Astrocytes express a neurotransmitter receptor called G-protein coupled metabotropic receptor (GPCR). Specifically, the G-protein G_q_, coupled to phospholipase C (PLC), is involved in elevating intracellular Ca^2+^ levels in astrocytes [91, 92]. When G_q_ is stimulated, PLC is activated to break down phosphatidylinositol 4,5-bisphosphate (PIP_2_) into inositol 1,4,5-triphosphate (IP_3_) and diacylglycerol (DAG) [91, 92]. By breaking down PIP_2_, the endoplasmic reticulum can release stored Ca^2+^ to stimulate gliotransmitter release.

In gliotransmission, astrocytes release vesicles that are packed with gliotransmitters via the process of exocytosis. Astrocytes express proteins that are known to be involved in vesicle fusion such as soluble NSF attachment protein receptor (SNARE), synaptotagmin, complexin2, and Munc18a, which are critical for gliotransmitter release [5, 92]. For example, altering the SNARE complex resulted in a failure of glutamate release from astrocytes [93]. Glutamate release also requires vacuolar type of proton ATPase to exchange the proton gradient from the vesicular lumen with glutamate [94, 95]. The same mechanism was observed for D-serine in hippocampal astrocytes [94]. In addition, in hippocampal astrocytes, synaptic-like microvesicles (SLMV) were found with the R-type SNAP receptor (R-SNARE), which is known to govern exocytosis [5]. Together, these proteins that are expressed by hippocampal astrocytes package gliotransmitters for release.

Additionally, ATP signaling regulates Ca^2+^-dependent glutamate release via astrocytic P2Y receptors [96]. ATP released from astrocytes also interacts directly with pre- and postsynaptic neurons, serving to regulate their own glutamatergic transmission and to also enhance the concentration of AMPA receptors, which facilitates the release of neuropeptides including oxytocin and vasopressin [65]. Additionally, some of the ATP released by astrocytes is converted directly to adenosine, which can act as both an agonist and antagonist for specific K^+^ and Ca^2+^ channels [97].

Cytokines and chemokine receptors are also implicated in the regulation of Ca^2+^ stores, glutamatergic transmission, and synaptic plasticity as a whole. In astrocytes, the CXCR4-CXCL12 signaling axis has been implicated in both modulating glutamate exocytosis, and in causing the release of the cytokine TNF-*α* [98]. TNF-*α* is also linked to regulating both glutamate release and the insertion of AMPA receptors into neighboring neurons [99, 100]. Finally, cytokine signaling in astrocytes, as well as microglia, plays a role in the response to physically sensing pain and responding to damage, with chemokine (C-C motif) ligand 2 (CCL2) released from astrocytes having a strong regulatory effect on the activity of NMDA receptors [101].

Despite the evidence indicating the significance of Ca^2+^ in the release of gliotransmitters, there have been controversial findings that challenge this assertion. Some studies have observed that blocking Ca^2+^ in hippocampal astrocytes located at the CA1 region *in situ* does not change Ca^2+^ levels in neurons, change spontaneous excitatory postsynaptic current, result in astrocytic glutamate release, or NMDA receptor mediated slow inward currents in pyramidal neurons [102–104]. These findings may suggest that a mechanism not dependent on Ca^2+^ release may lead to gliotransmitter release in astrocytes.

Although the gliotransmitters discussed above are important in regulating LTP, another crucial gliotransmitter to postsynaptic neurons is lactate. Memory formation is the result of a cascade of cellular and molecular processes and thus, to ensure the proper functionality of a neuron, astrocytes provide neurons with lactate, a usable form of energy [105–107]. Through glycogenolysis, astrocytes convert stored glycogen into lactate and release it into the synapse through the MCT1 or MCT4 transporter [107]. The neuron is then able to take up lactate via an MCT2 transporter, which has been confirmed through blocking MCT2 with either 4-CIN or MCT2-oligodeoxynucleotides [106, 107]. Rats showed memory impairment in inhibitory avoidance and spatial memory tasks when glycogenolysis, MCT1, MCT4, or MCT2 were inhibited [106, 107]. Thus, it is clear that the metabolism of astrocytes is critical in hippocampal dependent memory.

## 5. Ephrin Signaling and Glutamate Transporters

Ephrin signaling, consisting of ephrin-As and ephrin-Bs, is known for its involvement in neural development by inhibiting axonal and dendritic growth via actin rearrangement [108–114]. The interaction between ephrin-A3 and EphA4, which are expressed by astrocytes and dendritic spines of neurons, respectively, is involved in decreasing levels of GLAST and glutamate transporter 1 (GLT-1) for proper synapsing to occur [115–118].

Astrocytes express both EphB receptors and ephrin-B ligands, ephrinB3 being the most active during LTP [119]. EphrinB3 enhances D-serine release by regulating serine racemase (SR), an enzyme responsible for the conversion of L-serine to D-serine, and an SR-interacting protein, protein kinase C (PKC*α*). Specifically, ephrinB3 downregulates PKC*α* in order to increase the interaction between SR and Protein Interacting with C-kinase (PICK1), causing D-serine release [119]. Moreover, ephrinB3 is able to bind to both EphB3 and EphA4 receptors [120]. By measuring D-serine levels in EphB3 and EphA4 knockouts in cultured astrocytes, both receptors were necessary for D-serine release [119]. Thus, while ephrin-A signaling regulates levels of GLT-1, ephrin-B signaling regulates levels of D-serine release for activation of NMDA receptors.

## 6. Cholinergic Signaling

Nicotine influences memory by inducing synaptic transmission at acetylcholinergic synapses [121–123]. In Alzheimer's disease, patients treated with nicotine had improved cognitive functioning [124]. Astrocytes express nicotinic acetylcholine receptors (nAChR), implicating nicotine's role in cholinergic dependent memory. This effect on memory is dependent on glutamatergic NMDA receptors, which requires binding of D-serine released by astrocytes [81–83, 125]. As described previously, D-serine binds to NMDA receptors, allowing the influx of ions to induce LTP. Therefore, nicotine binding to the nAChR on astrocytes stimulates the release of D-serine by increasing internal calcium concentrations, allowing NMDA receptors on the postsynaptic neuron to induce LTP [123–125]. Similar to nAChR, activation of muscarinic AChR (mAChR) also increases internal calcium concentrations [126–128].

## 7. Other Receptors

### 7.1. Adenosine Receptors

There are other receptors thought to be involved in astrocyte-neuron communication. The adenosine A1 receptor is expressed on presynaptic neurons, and activation of the receptor activates the inhibitory metabotropic g-protein (G_i_) pathway. Memory deficits in mice that underwent 6 hours of sleep deprivation were prevented by pharmacologically blocking the A1 receptor [129]. Furthermore, astrocytes modulate levels of adenosine during 12 hours of sleep deprivation in mice [130]. Interestingly, A1 receptor activation in astrocytes can also modulate sleep in a rodent model of inflammation [131].

### 7.2. Interleukin-1

The cytokine interleukin-1 (IL-1) also plays a key role in hippocampal dependent memory. Blocking activity of IL-1 receptors resulted in the poor performance of learning with the Morris water maze and fear conditioning, as well as reduced LTP [132–135]. Although IL-1 receptors can be expressed by many cells, it is prominently expressed on astrocytes [136–140]. IL-1 receptor knockout mice that did not express IL-1 receptors on astrocytes exhibit memory deficits that can be rescued with transplantation of neural precursor cells from wild-type mice that express IL-1 receptors [139]. The underlying mechanism of IL-1 has yet to be determined in the context of memory.

## 8. Discussion

**Figure 1:**
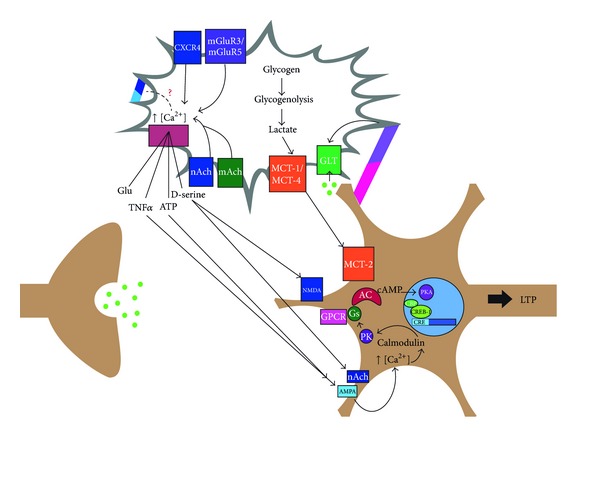
Integrative model of postsynaptic glia-neuron communication during memory formation.

Research on synaptic plasticity and memory has traditionally been neuron-centric, yet it is crucial to not ignore the astrocytic role in these processes since they are now known to modulate neuronal activity. Not only do astrocytes regulate the extracellular concentration of neurotransmitters, they also regulate the activity and expression of receptors on the postsynaptic neuron through gliotransmitter activity, and play a role in dampening activity and promoting the removal of nonadvantageous connections [141]. The evidence reviewed here shows that astrocytes have an ongoing role in the regulation of neuronal activity through the release of gliotransmitters and the expression of transporters/receptors on their extracellular surface. Based on these findings, we propose a mechanism of astrocyte-to-postsynaptic neuron interaction that supports the induction of LTP (see Figure 1). Here, the influx of intracellular Ca^2+^ caused by the activation of cholinergic receptors and mGluRs allows multiple gliotransmitters (e.g, glutamate, D-serine, TNF-*α*, and ATP) to be released. These gliotransmitters then bind to their respective receptor to regulate the influx of ions on the postsynaptic neuron, which causes a cascade of molecular mechanisms that initiate transcription. Ephrin B signaling may also contribute to gliotransmitter release by increasing intracellular Ca^2+^. Moreover, for the cellular and molecular changes of the postsynaptic neuron, lactate must be provided by astrocytes for energy to protect neurons from cytotoxic death, and GLT-1 regulates the extracellular glutamate concentration during the late phase of LTP.

The purpose of the proposed mechanism is to represent how astrocytes may regulate the postsynaptic neuron during LTP. Behavioral studies used to determine that the role of astrocytes are known to be hippocampal dependent tasks. However, this by no means allows us to determine which part of the hippocampus the mechanism takes place in, nor the type of LTP. More importantly, there are various kinds of memory such as episodic memory, procedural memory, associative memory, and fear conditioned memory. Moreover, it is important to note that this model only examines astrocyte to postsynaptic terminal communication: it is well known that astrocytes are also able to modulate presynaptic terminal [142, 143].

Although we have explained detailed evidence of how astrocytes regulate the postsynaptic neuron, we must also consider how astrocytes affect activity of the presynaptic neuron as well. Hippocampal astrocytes are able to detect synaptic activity at distinct locations via mGluR5 and increase intracellular Ca^2+^ levels for a prolonged time span, which results in alteration of basal synaptic transmission [144, 145]. The mechanism also involves astrocytic release of purines to activate A2A receptors expressed by the presynaptic neuron. Calcium activity was also observed to not only be involved in gliotransmission, but neurotransmission as well. Synaptic transmission in neighboring synapses was reduced when blocking Ca^2+^ in astrocytes, suggesting the ability of astrocytes to modulate the activity of presynaptic neurons.

The engraftment of human astrocytes in mice enhances LTP and significantly increases the release of the cytokine TNF-*α* [146]. Since a xenograft of human astrocytes can functionally modulate the activity of mice neurons, it may be possible that a xenograft from another species would facilitate LTP if placed into a human patient. There is still much to research in glial neurobiology in order to fully understand the underlying mechanisms of neural networks that are involved in plasticity and memory. For instance, since astrocytes are physically connected with other astrocytes through gap junctions to form a glial syncytium, it is crucial to further examine how astrocytic signaling may regulate neuronal activity and therefore, underlie LTP. It is now clear that astrocytes play an important part in learning and memory, and continuing to elucidate astrocytic processes that are involved in learning and memory will help advance our understanding of the dynamic role of these glial cells in modulating LTP.

## Figures and Tables

**Table 1:** Summary of receptors/signaling molecules and related mechanisms.

Signaling molecule	Receptor(s)	Mechanism of action in plasticity/memory formation
Acetylcholine	Muscarinic Ach-R's	Causes an increase in [Ca^2+^] activating mGluR's
Adenosine	A1 Receptors	Inhibition of cAMP dependent transcription
ATP	P2Y Receptors	Enhances concentration of AMPA receptors
Cytokines (i) TNF-*α* (ii) CCL2 (iii) Interleukin-1	(i) CXCR4 (ii) NMDA Receptors (iii) IL1 Receptors	(i) Glutamate release and the insertion of AMPA receptors (ii) Inhibits NMDA receptor activity (iii) Unknown
D-Serine	NMDA Receptors	Coagonist of receptors
Ephrin (i) Ephrin-A (ii) Ephrin-B	(i) EphA Receptors (ii) EphB Receptors	(i) Promotes retraction of dendritic spines (ii) Regulates D-serine release
Glutamate	AMPA receptors, NMDA receptors, mGluR's	Increased EPSP, upregulation of AMPA receptors
Lactate	MCT2	Provides additional metabolic energy for growth/plasticity
Nicotine	nAchR	Releases Ca^2+^ and promotes the release of D-serine
